# Supplementary material for: Modeling glioblastoma heterogeneity as a dynamic network of cell states
Source: Mol Syst Biol. 2021 Sep 16;17(9):e10105. doi: 10.15252/msb.202010105 (PMC8444284; doi:10.15252/msb.202010105)
Supplement: Supplementary file 6 — Source Data for Figure 5 [file MSB-17-e10105-s004.zip › Figure5A_sourcedata/GSEA_3071/hallmarks_stateII.GseaPreranked.1623742536692/HALLMARK_DNA_REPAIR.html]

Details for gene set HALLMARK\_DNA\_REPAIR[GSEA]

|  || Dataset | state23071 |
| Phenotype | NoPhenotypeAvailable |
| Upregulated in class | na\_pos |
| GeneSet | HALLMARK\_DNA\_REPAIR |
| Enrichment Score (ES) | 0.43886802 |
| Normalized Enrichment Score (NES) | 2.3936763 |
| Nominal p-value | 0.0 |
| FDR q-value | 1.8939395E-4 |
| FWER p-Value | 0.001 |
Table: GSEA Results Summary

  

Fig 1: Enrichment plot: HALLMARK\_DNA\_REPAIR      
 Profile of the Running ES Score & Positions of GeneSet Members on the Rank Ordered List

  

| PROBE | GENE SYMBOL | GENE\_TITLE | RANK IN GENE LIST | RANK METRIC SCORE | RUNNING ES | CORE ENRICHMENT || 1 | TYMS |  |  | 8 | 1.665 | 0.0388 | Yes |
| 2 | ZWINT |  |  | 16 | 1.510 | 0.0741 | Yes |
| 3 | FEN1 |  |  | 19 | 1.464 | 0.1095 | Yes |
| 4 | PCNA |  |  | 58 | 1.214 | 0.1298 | Yes |
| 5 | PRIM1 |  |  | 65 | 1.170 | 0.1570 | Yes |
| 6 | RFC3 |  |  | 67 | 1.157 | 0.1851 | Yes |
| 7 | POLD3 |  |  | 70 | 1.143 | 0.2127 | Yes |
| 8 | POLA2 |  |  | 95 | 1.068 | 0.2329 | Yes |
| 9 | RFC2 |  |  | 112 | 1.022 | 0.2540 | Yes |
| 10 | DUT |  |  | 118 | 1.009 | 0.2775 | Yes |
| 11 | RPA2 |  |  | 169 | 0.929 | 0.2878 | Yes |
| 12 | SAC3D1 |  |  | 188 | 0.887 | 0.3051 | Yes |
| 13 | RFC5 |  |  | 222 | 0.851 | 0.3178 | Yes |
| 14 | ALYREF |  |  | 315 | 0.741 | 0.3131 | Yes |
| 15 | LIG1 |  |  | 341 | 0.718 | 0.3246 | Yes |
| 16 | HPRT1 |  |  | 351 | 0.709 | 0.3397 | Yes |
| 17 | NT5C3A |  |  | 359 | 0.705 | 0.3553 | Yes |
| 18 | RFC4 |  |  | 385 | 0.686 | 0.3659 | Yes |
| 19 | PNP |  |  | 434 | 0.659 | 0.3701 | Yes |
| 20 | POLA1 |  |  | 443 | 0.654 | 0.3842 | Yes |
| 21 | SSRP1 |  |  | 478 | 0.638 | 0.3914 | Yes |
| 22 | POLH |  |  | 487 | 0.635 | 0.4050 | Yes |
| 23 | RAD51 |  |  | 506 | 0.627 | 0.4159 | Yes |
| 24 | RPA3 |  |  | 532 | 0.611 | 0.4247 | Yes |
| 25 | UPF3B |  |  | 536 | 0.610 | 0.4389 | Yes |
| 26 | SF3A3 |  |  | 711 | 0.530 | 0.4087 | No |
| 27 | POLD1 |  |  | 729 | 0.523 | 0.4173 | No |
| 28 | BCAM |  |  | 816 | 0.493 | 0.4081 | No |
| 29 | ADRM1 |  |  | 1034 | 0.432 | 0.3648 | No |
| 30 | NUDT21 |  |  | 1076 | 0.422 | 0.3650 | No |
| 31 | DGCR8 |  |  | 1122 | 0.414 | 0.3640 | No |
| 32 | DDB2 |  |  | 1189 | 0.399 | 0.3574 | No |
| 33 | IMPDH2 |  |  | 1228 | 0.391 | 0.3576 | No |
| 34 | POLE4 |  |  | 1281 | 0.381 | 0.3540 | No |
| 35 | NME1 |  |  | 1343 | 0.368 | 0.3479 | No |
| 36 | POLR2D |  |  | 1414 | 0.353 | 0.3392 | No |
| 37 | POLR3C |  |  | 1451 | 0.347 | 0.3388 | No |
| 38 | GTF3C5 |  |  | 1469 | 0.344 | 0.3430 | No |
| 39 | RAE1 |  |  | 1525 | 0.336 | 0.3376 | No |
| 40 | UMPS |  |  | 1923 | 0.282 | 0.2461 | No |
| 41 | TAF12 |  |  | 2030 | 0.269 | 0.2264 | No |
| 42 | CSTF3 |  |  | 2166 | 0.255 | 0.1991 | No |
| 43 | GTF2H1 |  |  | 2211 | 0.251 | 0.1944 | No |
| 44 | SURF1 |  |  | 2223 | -0.250 | 0.1978 | No |
| 45 | AK1 |  |  | 2446 | -0.276 | 0.1495 | No |
| 46 | XPC |  |  | 2518 | -0.288 | 0.1389 | No |
| 47 | NELFCD |  |  | 2642 | -0.307 | 0.1160 | No |
| 48 | APRT |  |  | 2680 | -0.313 | 0.1145 | No |
| 49 | DCTN4 |  |  | 2686 | -0.314 | 0.1209 | No |
| 50 | PDE4B |  |  | 2788 | -0.333 | 0.1040 | No |
| 51 | RNMT |  |  | 2805 | -0.336 | 0.1083 | No |
| 52 | POLD4 |  |  | 2936 | -0.362 | 0.0849 | No |
| 53 | RRM2B |  |  | 2940 | -0.363 | 0.0931 | No |
| 54 | VPS37B |  |  | 3099 | -0.398 | 0.0636 | No |
| 55 | SUPT5H |  |  | 3181 | -0.424 | 0.0539 | No |
| 56 | NME4 |  |  | 3252 | -0.442 | 0.0474 | No |
| 57 | SDCBP |  |  | 3257 | -0.442 | 0.0573 | No |
| 58 | TAF10 |  |  | 3313 | -0.461 | 0.0549 | No |
| 59 | MPG |  |  | 3321 | -0.464 | 0.0646 | No |
| 60 | EIF1B |  |  | 3420 | -0.509 | 0.0527 | No |
| 61 | COX17 |  |  | 3461 | -0.524 | 0.0556 | No |
| 62 | GUK1 |  |  | 3479 | -0.533 | 0.0645 | No |
| 63 | ARL6IP1 |  |  | 3574 | -0.586 | 0.0555 | No |
| 64 | NCBP2 |  |  | 3588 | -0.598 | 0.0670 | No |
| 65 | VPS28 |  |  | 3643 | -0.639 | 0.0692 | No |
| 66 | NME3 |  |  | 3783 | -0.772 | 0.0537 | No |
| 67 | GPX4 |  |  | 3915 | -0.986 | 0.0454 | No |
Table: GSEA details [plain text format]

  

Fig 2: HALLMARK\_DNA\_REPAIR: Random ES distribution      
 Gene set null distribution of ES for **HALLMARK\_DNA\_REPAIR**
